# Feasibility Study to Byproduce Medical Radioisotopes in a Fusion Reactor

**DOI:** 10.3390/molecules28052040

**Published:** 2023-02-22

**Authors:** Jia Li, Shanliang Zheng

**Affiliations:** 1 School of Nuclear Science and Technology, University of Science and Technology of China, Hefei 230000, China; 2 Institute of Plasma Physics, Chinese Academy of Sciences, Hefei 230000, China

**Keywords:** medical radioisotopes, fusion reactor, yields, specific activity

## Abstract

Currently, international nuclear fission reactors producing medical isotopes face the problem of shutdown and maintenance, decommissioning, or dismantling, while the production capacity of domestic research reactors for medical radioisotopes is inadequate, and the supply capacity for medical radioisotopes faces major challenges in the future. Fusion reactors are characterized by high neutron energy, high flux density, and the absence of highly radioactive fission fragments. Additionally, compared to fission reactors, the reactivity of the fusion reactor core is not significantly affected by the target material. By building a preliminary model of the China Fusion Engineering Test Reactor (CFETR), a Monte Carlo simulation was performed for particle transport between different target materials at a fusion power of 2 GW. The yields (specific activity) of six medical radioisotopes (^14^C, ^89^Sr, ^32^P, ^64^Cu, ^67^Cu, and ^99^Mo) with various irradiation positions, different target materials, and different irradiation times were studied, and compared with those of other high-flux engineering test reactors (HFETR) and the China Experimental Fast Reactor (CEFR). The results show that this approach not only provides competitive medical isotope yield, but also contributes to the performance of the fusion reactor itself, e.g., tritium self-sustainability and shielding performance.

## 1. Introduction

There are more than 100 types of radioisotopes used in medicine worldwide, including more than 30 medical radioisotopes used for the diagnosis and therapy of diseases. The diagnostic (imaging) medical isotopes emit mainly γ-rays or positrons, and preferably no α and β-particles, with a γ energy range of 100–511 keV being appropriate. Therapeutic medical isotopes mainly emit alpha and beta particles, internal conversion electrons, and Auger electrons, instead of emitting gamma rays or fewer. The energy of beta particles should be below 1 MeV, and that of alpha particles should be below 6 MeV [1].

The principle of medical radioisotope production is based on bombarding the nucleus of a stable isotope with particles (e.g., neutrons, protons), causing a nuclear reaction that transforms the stable nuclide into an unstable radioactive nuclide. The three main methods of producing medical radioisotopes are reactors, particle accelerators, and nuclide generators. In reactor production, a distinction can be made between activation by irradiation and extraction of fission products; most of the commonly used artificial radionuclides are produced by extracting fission products from the reactor. Nevertheless, most of these reactors have been in operation for more than 40 years and are facing shutdown, decommissioning, or dismantling, and there is also a risk of nuclear proliferation. There are five main research reactors for medical radioisotope production in China, which are listed in Table 1. in the literature [2,3]. Except for the HFETR (High-Flux Engineering Test Reactor) and CMRR (China Mianyang Research Reactor), which can produce ^131^I and ^89^Sr in small quantities (meeting only 20% of domestic needs), these reactors are not capable of producing medical radioisotopes in large quantities. In addition, no medical radioisotopes have been produced in commercial fission reactors [2]. The most urgent need is for ^99^Mo/^99m^Tc (^99m^Tc is mainly obtained by the “Mo-Tc generator”), and the current weekly demand for ^99^Mo in medicine is approximately 12,000 Ci (6 days Ci), which can be used approximately 40 million times per year and accounts for 80% of clinical SPECT imaging drugs [4].

Accelerators, such as linacs and cyclotrons, are mainly used for the production of positron-like radionuclides, such as ^18^F and other short-lived nuclides; however, most of the current medical cyclotrons in China rely on imports from abroad, and nuclide yields are much lower than reactor production due to factors such as beam intensity and nuclear reaction cross-section [5]. A recent International Atomic Energy Agency (IAEA) study of global radioisotope production found that although medical radioisotope production continues, transportation and distribution problems due to the COVID-19 epidemic are causing serious shortages of medical isotopes in the short term, and that, in the long term, supply capacity for medical radioisotopes also faces major challenges [6].

This paper focuses on a feasibility study of producing medical isotopes based on fusion reactors. With the development of new high-temperature superconducting technology (HTS), fusion energy could soon be commercially exploited [7]. We hope to take full advantage of the properties of the fusion reactor—high neutron energy, broad distribution of the energy spectrum, high particle flux density, no production of radioactive fission fragments, and high safety (low risk in relation to nuclear proliferation)—and to select the nuclide species suitable for fusion reactor production. In this way, a new possibility to solve the shortage of medical radioisotopes is offered.

## 2. Results and Discussion

### 2.1. Results

Based on the target materials listed in the Section 3.1, and assuming that the targets are irradiated for 365 days at 2.0 GW fusion power with one-hour cooling, the yield per unit mass of the target (GBq/g) is first calculated for target zones #1~#3, and the simulation results are shown in Table 1. Assuming that the irradiation time is one year, the initial goal is to achieve a saturated yield for each radioisotope (except ^14^C). The optimized calculation and analysis of the irradiation time and target composition for each medical isotope is then presented in Section 2.2.

### 2.2. Discussion

#### 2.2.1. ^14^C

^14^C has a half-life of 5700(30) a and is a decaying low-energy electron radionuclide used mainly for in vitro detection of radioactive markers. Therefore, ^14^C is considered the gold standard for H.Pylori (Hp) testing and is widely used in clinical practice [8,9].

^14^C is generated by the ^14^N (n, p) reaction, whose cross-section increases with decreasing energy. Table 1 shows that the ^14^C production is proportional to the total neutron flux density in the target region; the ^14^C yields for one year of irradiation at full power (2.0 GW) at the CFETR in TAR 1 are comparable to the yields (3.80 × 10^7^ Bg/g) for a lifetime of 2000 MW-d (corresponding to an irradiation time of ~28 days) at the high-flux experimental reactors HFETR [10]. This means that the efficiency of ^14^C production in the fusion reactor is significantly lower than in the HFETR. Table 2 compares the effects of different irradiation times on the yield in the CFETR. Due to the very long half-life of ^14^C, the longer the irradiation time is, the higher the yield, and that 10-year irradiation is an order of magnitude higher than one-year irradiation. Therefore, the target material should be in the place where the shielding material is located. It would work well for both ^14^C production and thermal neutron absorption.

#### 2.2.2. ^89^Sr

^89^Sr has a half-life of 50.563 (25) d and emits mainly β-rays (branch ratio 99.9905%) with a maximum energy of 1.501MeV, while another branch (branch ratio 0.0095%) also undergoes β-decay to produce ^89m^Y and emits γ-rays with an energy of 0.909 MeV through IT decay. ^89^Sr, as a typical therapeutic radionuclide, is mainly used for the treatment of bone tumors [11].

There are the two methods for the preparation of ^89^Sr, ^88^Sr(n, γ) and ^89^Y(n, p) reactions. It was found that full-power irradiation of Sr_2_CO_3_ with the CFETR for one year compared to the typical yield of 56 days of irradiation with the HFETR (~0.29 Ci/g) [12], the maximum yield in TAR1 (1.16 × 10^1^ GBq/g ≈ 0.31 Ci/g) has no appreciable advantage, and since the (n, γ)-reaction tends to produce ^85^Sr, ^83^Sr, and other radioactive impurities, the ^85^Sr yield is high and the half-life is long (64.86 d). It is impossible to remove it by radiochemical separation. This would significantly affect the radionuclide purity (RNP) of the final ^89^Sr product. Therefore, the production of ^89^Sr by the ^88^Sr(n, γ) reaction generally requires the use of Sr_2_CO_3_ targets with high enrichment (^88^Sr > 99.99%), which would increase production costs.

The other production method (n, p) has a reaction threshold and is generally pro-duced in fast neutron reactors. The results from the literature [13,14] show that the yield of ^89^Sr is ~0.011 Ci/g for 30 days of continuous irradiation with natural Y_2_O_3_ in a fast-breeder test reactor (FBTR), while the yield of TAR 1 reaches 0.12 Ci/g (4.44 GBq/g) for 30 days of full power irradiation in the CFETR; it is ~10 times higher than that in a FBTR, and the yield can be further increased if the irradiation time is extended. Table 3 shows the variation of ^89^Sr yield with irradiation time: the yield in the CFETR can reach 0.293 Ci/g (1.08 × 10^1^ GBq/g) at 200 days of irradiation, which is essentially saturated; while in the China Experimental Fast Reactor (CEFR), the typical yield is 0.0119 Ci/g at two refueling cycles (~180 days) [15]. It should be noted that fast neutrons also trigger ^89^Y (n, 2n)^88^Y reactions with high yields, e.g., the ^88^Y yield reaches 0.005 Ci/g at 30-day irradiation in a FBTR [13]; the ^88^Y radionuclide impurity (T_1/2_ = 106.65 d) is susceptible to (EC, β+) decay and concomitant gamma decay, so the reprocessing pathway must be remotely controlled to ensure radiation safety.

The ^88^Sr(n, γ) method to produce ^89^Sr requires a highly enriched ^88^Sr target material, which is costly, and the yield advantage is not significant compared to high-flux reactors (e.g., the HFETR). In contrast, ^89^Sr produced by the (n, p) reaction is carrier-free and the target is natural Y_2_O_3_ without enrichment treatment. The yield of ^89^Sr in the CFETR is significantly higher than that of other high-flux reactors (e.g., the HFETR) and experimental fast reactors (e.g., the FBTR and the CEFR), due to the advantages of high neutron energy and high neutron flux density.

#### 2.2.3. ^32^P

^32^P has a half-life of 14.3 days and decays only β-rays; it is a common therapeutic radionuclide and is mainly used in clinical practice for the treatment of primary thrombocythemia and for the topical treatment of skin diseases such as hemangiomas. In addition, radioactive stents made of ^32^P can prevent restenosis after coronary angioplasties, and can also relieve bone metastasis pain [16].

^32^P is produced in a similar way to ^89^Sr, also by (n, γ)- and (n, p)-reactions. The first method is produced by a neutron capture reaction using natural phosphorus (^31^P abundance 100%) as the target material. The ^32^P obtained by this method is not carrier-free, and the yield is low, due to the small ^31^P-(n, γ)^32^P reaction cross-section, but this method can obtain a noncarrier-free sodium phosphoric acid [^32^P] solution by simple chemical post-treatment, and it is the preferred method for the preparation of ^32^P in high-flux thermal neutron reactors [17]. In the second method, natural sulfur (^32^S 95.05%, ^33^S 0.75%, ^34^S 4.21%) is used as the target material and produced by the ^32^S(n, p)^32^P reaction, which is suitable for production in fast reactors; the reaction cross-section is higher than the cross-section for thermal neutron capture, and the specific activity yield is high and carrier-free. The results in Table 1 show that the yield of the (n, p) reaction is an order of magnitude higher than that of the (n, γ) reaction for the same target. Compared with the typical yields for ^32^P via ^31^P(n, γ) at the HFETR (0.79 Ci/g) [17], and via ^32^S(n,p) at the CEFR (13.1 Ci/g) [15], the yield of TAR 1 at the CFETR (20.4 Ci/g ≈ 7.55 × 10^2^ GBq/g, 90 days irradiation) is more significant.

However, the main disadvantage of the ^32^S (n, p)^32^P method is that the subsequent radiochemical treatment is complex and very time-consuming [18]. In addition, the ^33^S (n,p)^33^P reaction produces the radioactive impurity ^33^P (T_1/2_ = 25.34 d), but its low yield (three orders of magnitude lower than yield of ^32^P) has negligible effects on the radionuclide purity (RNP) of ^32^P. Table 4 calculates the variation in ^32^P yield as a function of irradiation time and shows that the yield essentially saturates when the irradiation time reaches 90 days.

#### 2.2.4. ^64^Cu, ^67^Cu

^64^Cu has a half-life of 12.7 h and can undergo low-energy positron decay (branching ratio 17.5%), as well as β-decay (38.5%) and EC–decay (44.0%), so it can be used for PET imaging as well as radioimmunotherapy. ^67^Cu has a half-life of 2.58 days and decays mainly low-energy β-rays (maximum electron energy 577 keV) and is a promising theranostic radionuclide [19].

It is common to use a proton cyclotron to produce ^64^Cu and ^67^Cu, for example, ^64^Cu by a ^64^Ni(p, n) reaction using a small medical cyclotron (proton energy 14–9 MeV) irradiating a 96% enriched ^64^Ni target with a saturation yield of 159 mCi (5.88 GBq)/μA, which is equivalent to 5.90 × 10^2^ GBq/g (proton energy 11.4 MeV, beam intensity 30 μA, irradiation 8 h) [20]. ^67^Cu is generally produced with intermediate energy protons to irradiate highly enriched ^68^Zn targets by the ^68^Zn(p, 2p)^67^Cu reaction, with a typical yield of approximately 24.3 MBq/μA∙h, which is equivalent to 3.8 GBq/g (70–35 MeV protons, beam intensity 30 μA, irradiation 24 h). The photonuclear production for ^67^Cu using bremsstrahlung photons with an eLINAC accelerator has also been studied for decades. Recently, a large enriched ^68^Zn target (55.5 g) was irradiated with 40 MeV e-LINAC for 53.5 h, obtaining 62.9 GBq (1.7 Ci) without detecting ^64^Cu [19].

However, all of the above approaches require enriched target materials, which has not only economic implications for the initial investment, but also technological implications for radiochemical processing and target recovery.

In recent years, a route involving irradiation of natural ZnO targets with fast neutrons by (n, p) reactions has also been proposed [21], with higher reaction thresholds for the ^64^Zn(n, p)-^64^Cu and ^67^Zn(n, p)-^67^Cu reactions, as listed in Section 3.1. In the literature [21], the above two reaction channels were compared in the fission spectrum and neutron scattering spectra of 14-MeV deuterium(D)-beryllium (Be), and the average reaction cross-section of the fast neutron spectra is 3 to 5 times higher than that of the fission spectrum. It can be seen that a sufficiently “hard” neutron energy and a sufficiently high neutron flux are required for the production of ^64^Cu and ^67^Cu, and that the fusion reactor has advantages in these two aspects. From the yield results in Table 1, the highest yield of ^64^Cu in TAR 1 reached 89.5 GBq/g (saturation value can be reached after 3 days of irradiation), and the highest yield of ^67^Cu reached 1.92 GBq/g (saturation value can be reached after 10 days of irradiation), which is lower than the yield of ^64^Cu and ^67^Cu produced by proton irradiation for enriched-target. Since the abundance of ^67^Zn is only 4.04% and ^64^Zn accounts for 49.17% of natural zinc, this also results in a low RNP of ^67^Cu. When ^nat^ZnO is replaced by enriched ^64^Zn (100%) and enriched ^67^Zn (100%), the yield of ^64^Cu in TAR1 can reach 214 GBq/g, while the yield of ^67^Cu in TAR1 will reach 40.5 GBq/g.

On the other hand, if some ^64/67^Cu-radiopharmaceuticals are acceptable for clinical application, irradiation of ^nat^ZnO targets using the fusion reactor seems to be a better option, since it provides a higher ^64/67^Cu yield at a lower price per GBq, in comparison with proton irradiation and photonuclear production.

#### 2.2.5. ^99^Mo

^99^Mo (T_1/2_ = 66 h) is used as the parent nuclide of ^99m^Tc, mainly for the production of “molybdenum-technetium (^99^Mo-^99m^Tc) generators”. ^99^Mo is produced in the fusion reactor mainly through three reaction channels (see Section 3.1): the (n, γ)-reaction cross-section for thermal neutrons is about 130 mb, which is much lower than the effective reaction cross-section of ^99^Mo produced by ^235^U fission (37 b); ^100^Mo(n, 2n) ^99^Mo has a reaction threshold (8.4 MeV), with a maximum reaction cross-section of about 1.5 b when the fast neutron energy is 14 MeV [22]; ^99^Mo via ^100^Mo(γ, n) is produced by the photonuclear reaction, where the reaction threshold is 9.1 MeV and the maximum reaction cross-section is ~150 mb when the photon energy is 14.5 MeV [23].

Table 5 shows the ^99^Mo yields of irradiated natural molybdenum for different irradiation times. Since the half-life of ^99^Mo is about 66 h, the optimum irradiation time is 8 days (about three half-lives), and the saturation yield is reached at 21 days of irradiation. There is almost no difference between the yields at one-year and 21-day irradiation.

The yields from two highly enriched molybdenum (100% enriched ^100^Mo and 100% enriched ^98^Mo) were compared with those from natural molybdenum (^92^Mo 14.84%, ^94^Mo 9.25%, ^95^Mo 15.92%, ^96^Mo 16.68%, ^97^Mo 9.55%, ^98^Mo 24.13%, ^100^Mo 9.63%), as shown in Table 6. The yield from ^100^Mo target is highest in TAR 1 mainly by (n, 2n) reaction, as this region has the highest fraction of fast neutrons; in TAR 2, the yield from ^nat^Mo is higher than that of ^100^Mo, but lower than that of ^98^Mo; the yield from ^98^Mo is highest in TAR3 mainly by (n, γ) reaction, where the fraction of thermal neutrons is highest.

Under ideal simulation conditions, the maximum yield of ^99^Mo can reach a specific activity of 24.6 Ci/g (9.10 × 10^2^ GBq/g) in TAR1, which is still three orders of magnitude lower than the typical yield of ^99^Mo from fission products in fission reactors (10,000 Ci/g), although it is comparable to the optimal yield of proton or electron accelerators (~10 Ci/g) and can be used in low specific activity ^99^Mo-^99m^Tc generators [24].

## 3. Materials and Methods

### 3.1. Principle of Reaction

The principle of radionuclide production by a fusion reactor is essentially based on the following four reactions:

1. XZA(n,γ)XZA+1 reaction: Target nucleus XZA captures a neutron and becomes a nucleus in an excited state X*ZA, which is immediately deexcited to return to the ground state, accompanied by the release of gamma rays.
2. XZA(n,p)YZ−1A reaction: The target nucleus captures a neutron and immediately releases a proton, and the target nucleus and the daughter nucleus no longer belong to the same chemical element. In general, the (n, p) reaction requires high neutron energy and has a reaction threshold, but there are special cases, such as ^14^N(n,p)^14^C, whose reaction cross-section is high in the thermal neutron energy range [8].
3. XZA(n,2n)XZA−1 reaction: Fast neutron interaction via the inelastic reaction. Usually, has a reaction energy threshold.
4. XZA(γ,n)XZA−1 reaction: Neutron photo-production. Usually, has a reaction energy threshold.

Considering the above reaction characteristics and fusion reactor properties, a feasibility study on the production of six medical radioisotopes, ^14^C, ^89^Sr, ^32^P, ^64^Cu, ^67^Cu, and ^99^Mo, using fusion reactors is proposed. Their reaction channels and corresponding decay modes are listed in Table 7.

### 3.2. Computational Models and Simulation Tools

**Figure 1:**
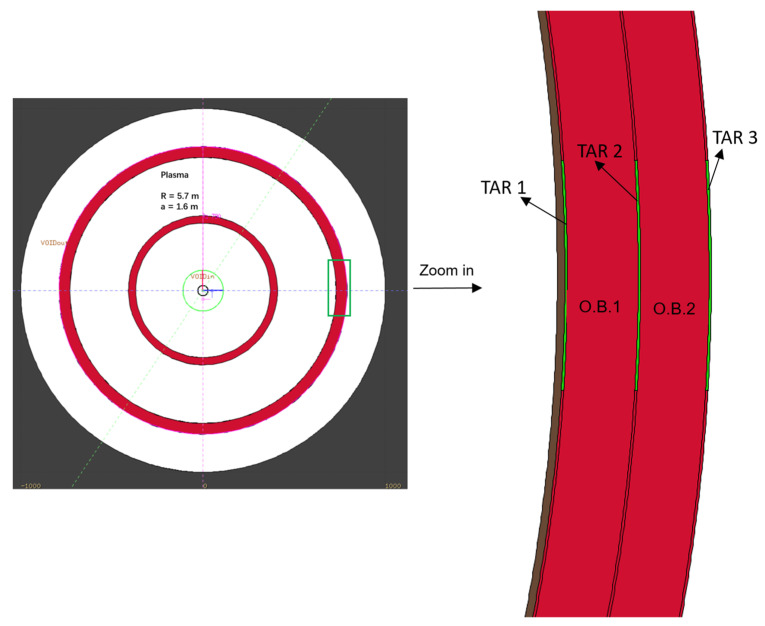
One-dimensional CFETR model (FLUKA modelling).

To preliminarily estimate the feasibility and the effectiveness of producing medical radioisotopes in a fusion reactor, a one-dimensional spherical model was built to represent the CFETR [25], where the main radius is R = 5.7 m and the minor radius is a = 1.6 m (Figure 1). The radial size and material of each component are listed in Table 8, with “I.B.” and “O.B.” denoting the inner and outer breeding blanket zones. The breeding blanket and first wall are the primary components responsible for extracting fusion power, generating tritium fuel, and protecting against radiation.

The targets in the outboard breeding blanket (as shown in Figure 1) are positioned near the equatorial port where diagnostic systems would be located. This location was chosen to make it easier to install and remove the target bulk, allowing for the extraction of any medical radioisotopes produced. The radial thickness of each target was set as 1 cm to avoid excessive heat deposition; such a thin target would not introduce significant impact on the neutron shielding and scattering in the neighborhood, so there is no impact on the primary functions of the fusion reactors.

The production capacity of radioisotopes in a fusion reactor is greatly influenced by the internal nuclear environment. To initially estimate the production capacity of the CFETR, the one-dimensional radiation transport simulation with integral activation calculations has been employed to characterize the radiation environment. This approach is common in nuclear analysis and provides a baseline for further optimization of the production of medical radioisotopes in fusion reactors. However, the one-dimensional model may underestimate the particle flux, as it does not account for radiation streaming through ports and penetrations in the blanket, as well as the complex geometry of the tokamak system [26]. To obtain a more accurate estimate of the production capacity, an additional three-dimensional CFETR model will be constructed in future research, taking into account these factors.

The particle transport and material activation in the simulation were performed using the FLUKA program developed by CERN [27]. The latest version (4–3.1) of FLUKA features a point-wise interaction model for low-energy neutrons (below 20 MeV), which was activated using the LOW-PWXS card. The ENDF-VIII.0 library was used as the source of low-energy neutron cross-sections. The induced radioactivity calculation was activated using the RADDECAY card. The program also includes parametrized cross-sections for photonuclear interactions, based on a combination of experimental data and theoretical models, which was activated using the PHOTONUC card.

**Figure 2:**
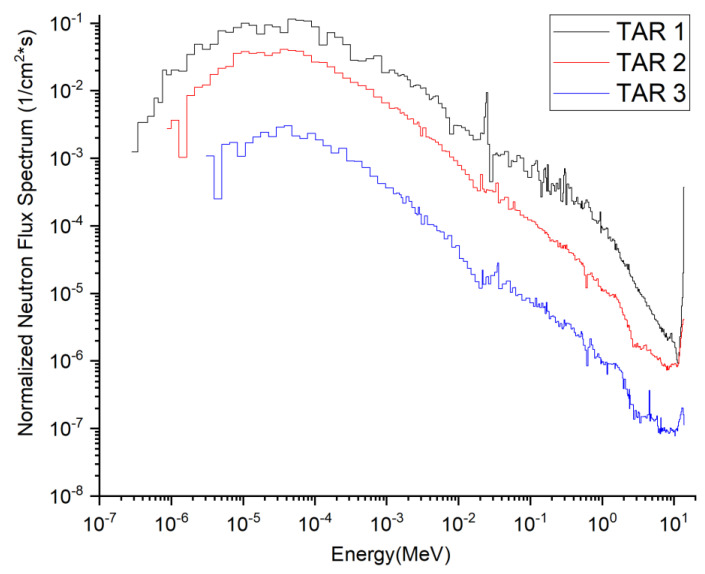
Total flux density spectra of neutrons at the incident surfaces of the three target segments.

**Figure 3:**
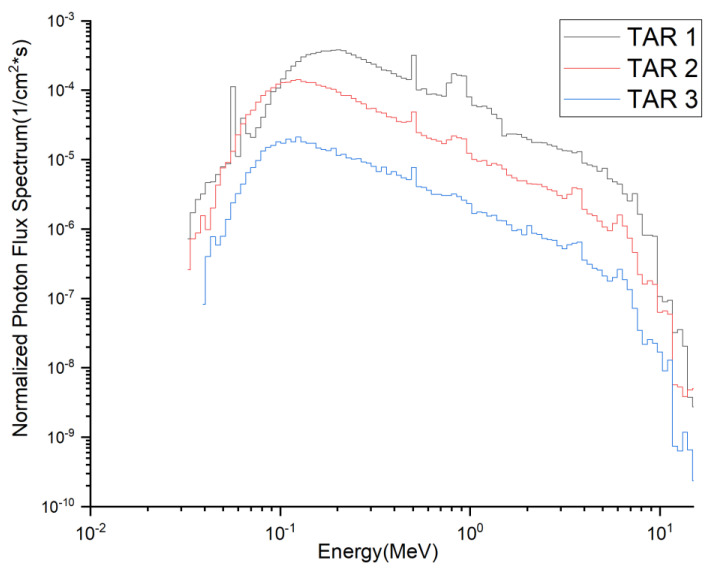
Total flux density spectra of photons at the incident surfaces of the three target segments.

The targets to be irradiated and the corresponding medical isotopes produced are listed in Table 7. The composition of the targets was chosen mainly with reference to the corresponding materials produced for that medical isotope in other reactors or accelerator facilities, to allow the comparison of typical yields. The total flux density spectra of neutrons and photons at the incident surfaces of the three target segments are shown in Figure 2 and Figure 3. The flux density spectra are normalized to a single source neutron per unit area of the target (unit: 1/cm^2^·s). The total neutron flux densities (Φ_n_) and photon flux densities (Φ_p_) at these three interfaces are listed in Table 9, all multiplied by the neutron source strength (7.10 × 10^20^ n/s) at 2.0 GW fusion power. The corresponding average neutron energies $\bar{E_{n}}$ and average photon energies $\bar{E_{p}}$ were also calculated.

## 4. Summary

Fusion reactors are characterized by high neutron energy, high flux density, and the absence of highly radioactive fission fragments. Additionally, compared to fission reactors, the reactivity of the reactor core is insensitive to the influence of the target material.

Therefore, fusion reactors are suitable for the production of therapeutic medical radioisotopes with large (n, p) cross-sections of fast neutrons, e.g., ^89^Sr, ^64^Cu, ^67^Cu, and ^32^P, and the products of the (n, p) reaction mode are “carrier-free” compared to the thermal neutron capture reaction (n, γ), resulting in a higher specific activity. The effects of the placement of these target materials and the size of the targets on the performance of the fusion reactor need further study.

Optimal ^99^Mo production in a fusion reactor should be chosen in the region of high neutron flux and neutron energy, e.g., at the back of the first wall and at the front of the breeding blanket. This is because the ^100^Mo(n, 2n)^99^Mo reaction not only provides the competitive ^99^Mo yield, but also facilitates the multiplication of neutrons to increase the tritium yield of the breeding blanket, and thus improve the tritium self-sustainability of the fusion reactor.

The advantage of ^14^C production in fusion reactors is not significant, but due to the high thermal neutron cross-section of the (n, p) reaction and the sufficiently long half-life of ^14^C, the AlN target can be placed as a shielding material in front of the shielding blanket to irradiate as long as possible, to achieve a good effect in thermal neutron absorption while allowing additional ^14^C production.

Table 10 summarizes and compares the typical yields of the six medical isotopes in the CFETR and other reactor types or accelerators. The highest neutron flux density of the CFETR is 5.17 × 10^14^ at TAR 1; the average thermal neutron flux density of the HFETR is 1.1 × 10^14^; while the highest flux densities of fast neutrons (E ≥ 0.1 MeV) of the CEFR and the FBTR are 2.5 × 10^15^ and 2.45 × 10^15^, respectively.

In a further study, the position and thickness of the irradiation target can be further optimized according to the nuclear-thermal analysis and the operation parameters of the 3D CFETR model, and a corresponding radiation-protection plan for the subsequent radiochemical process can be proposed from the radiation safety point of view.

## Figures and Tables

**Table 1:** Yield of medical radioisotopes from TAR1 to TAR3 at a fusion power of 2.0 GW, an irradiation time of 365 d, and a cooling time of 1 h.

Target	Yields (GBq/g)							
	^14^C	^99^Mo	^88^Sr(n, γ)^89^Sr	^89^Y(n, p)^89^Sr	^31^P(n, γ)^32^P	^32^S(n, p)^32^P	^64^Cu	^67^Cu
TAR 1	3.21 × 10^−2^	2.92 × 10^2^	1.16 × 10^1^	1.28 × 10^1^	3.12 × 10^1^	7.72 × 10^2^	8.95 × 10^1^	1.92
TAR 2	4.58 × 10^−3^	5.46 × 10^1^	1.85	7.51 × 10^−1^	5.53	8.49 × 10^1^	8.81	1.11 × 10^−1^
TAR 3	2.79 × 10^−4^	2.19	7.37 × 10^−2^	4.47 × 10^−2^	3.62 × 10^−1^	7.39	6.79 × 10^−1^	5.61 × 10^−3^

**Table 2:** ^14^C yields of AlN at various irradiation times.

Target	^14^C Yields (Bq/g AlN)			
	1 d	28 d	1 Year	10 Years
TAR 1	8.80 × 10^4^	2.46 × 10^6^	3.21 × 10^7^	3.20 × 10^8^
TAR 2	1.25 × 10^4^	3.51 × 10^5^	4.58 × 10^6^	4.62 × 10^7^
TAR 3	7.62 × 10^2^	2.14 × 10^4^	2.79 × 10^5^	2.62 × 10^6^

**Table 3:** ^89^Sr yields of Y_2_O_3_ at various irradiation times (days).

Target	^89^Sr Yields (GBq/g Y_2_O_3_)					
	30 d	50 d	100 d	150 d	200 d	365 d
TAR 1	4.44	6.40	1.06 × 10^1^	1.07 × 10^1^	1.08 × 10^1^	1.28 × 10^1^
TAR 2	2.28 × 10^−1^	3.66 × 10^−1^	5.74 × 10^−1^	5.88 × 10^−1^	5.88 × 10^−1^	7.51 × 10^−1^
TAR 3	9.73 × 10^−3^	2.27 × 10^−2^	3.81 × 10^−2^	3.92 × 10^−2^	3.92 × 10^−2^	4.48 × 10^−2^

**Table 4:** ^32^P yields of ^nat^S at various irradiation times (days).

Target	^32^P Yields (GBq/g ^nat^S)			
	30 d	60 d	90 d	365 d
TAR 1	5.88 × 10^2^	7.25 × 10^2^	7.55 × 10^2^	7.73 × 10^2^
TAR 2	6.44 × 10^1^	7.96 × 10^1^	8.29 × 10^1^	8.29 × 10^1^
TAR 3	5.29	6.51	6.81	6.99

**Table 5:** ^99^Mo yields of ^nat^Mo at various irradiation times (days).

Target	^99^Mo Yields(GBq/g ^nat^Mo)			
	1 d	8 d	21 d	365 d
TAR 1	6.56 × 10^1^	2.55 × 10^2^	2.92 × 10^2^	2.94 × 10^2^
TAR 2	1.22 × 10^1^	4.77 × 10^1^	5.46 × 10^1^	5.49 × 10^1^
TAR 3	4.90 × 10^−1^	1.91	2.19	2.19

**Table 6:** ^99^Mo yields with various enrichments of molybdenum isotope at a fusion power of 2.0 GW, 21-day irradiation.

Target	^99^Mo Yields (GBq/g)		
	^nat^Mo	^100^Mo (100%)	^98^Mo (100%)
TAR 1	2.92 × 10^2^	9.10 × 10^2^	7.74 × 10^2^
TAR 2	5.46 × 10^1^	4.96 × 10^1^	1.88 × 10^2^
TAR 3	2.19	3.59	6.81

**Table 7:** Target material and corresponding radioisotopes.

Nuclide	Decay Mode	Reaction Channel	Threshold (Yes/No)	Target Material	Application
^14^C	β^−^	^14^N(n, p) ^14^C	No	AlN	Testing for H.Pylori (Hp)
^89^Sr	β^−^	^88^Sr(n, γ) ^89^Sr	No	SrCO_3_	Therapeutic radionuclides
		^89^Y(n, p)^89^Sr	Yes (720 keV)	Y_2_O_3_	
^32^P	β^−^	^31^P (n, γ) ^32^P ^32^S (n, p) ^32^P	No	^nat^P	Therapeutic radionuclides
			Yes (957 keV)	^nat^S	
^67^Cu	β^−^	^67^Zn(n, p) ^67^Cu	Yes (3.0 MeV)	^nat^ZnO	Novel Targeted Therapeutic radionuclides
^64^Cu	β^+^, (β^−^, EC)	^64^Zn(n, p)^64^Cu	Yes (0.9 MeV)	^nat^ZnO	PET imaging, Immunotherapy
^99^Mo	β^−^	^98^Mo(n, γ) ^99^Mo	No	^nat^Mo Enriched-Mo	Mo-Tc generator
		^100^Mo(n, 2n) ^99^Mo	Yes (8.4 MeV)		
		^100^Mo(γ, n) ^99^Mo	Yes (9.1 MeV)		

**Table 8:** Radial dimensions and material arrangement.

ID	Inner Radius (cm)	Outer Radius (cm)	Zone Name	Mat. Vol. Ratio (%)
1	370	410	I.B.	Be_12_Ti (65.6), Li_2_TiO_3_(14.4)
2	410	730	Plasma	vacuum
3	730	733	First wall	EUROFER (60), vapour (40)
4	733	734	TAR 1	-
5	734	762	O.B. 1	Be_12_Ti (65.6), Li_2_TiO_3_(14.4)
6	762	763	TAR 2	-
7	763	790	O.B. 2	Be_12_Ti (65.6), Li_2_TiO_3_(14.4)
8	790	791	TAR. 3	-

**Table 9:** Total neutron/photon flux densities and average neutron/photon energies at the incident surfaces of the three target segments.

Zone	Φ_n_ (n/cm^2^.s)	$\bar{E_{n}}$ (MeV)	Φ_p_ (p/cm^2^.s)	$\bar{E_{p}}$ (MeV)
TAR 1	5.17 × 10^14^	2.73	1.86 × 10^14^	1.18
TAR 2	8.24 × 10^13^	4.37 × 10^−1^	4.45 × 10^13^	1.44
TAR 3	5.39 × 10^12^	6.44 × 10^−1^	7.02 × 10^12^	1.58

**Table 10:** Typical yield comparison of CFETR with other types of reactors and accelerators.

Nuclide	T_1/2_	CFETR		Other Reactors or Accelerators		
		Irradiation Time	Typical Yields (GBq/g)	Typical Yields (GBq/g)	Irradiation Parameter	Reactor (N_flux n/cm^2^·s) or Accelerator (Beam Parameters)
^14^C	5730a	28 d	2.46 × 10^−3^	3.85 × 10^−2^	28 d	HFETR (1.1 × 10^14^)
		365 d	3.21 × 10^−2^	5.00 × 10^−1^	365 d	
^89^Sr	50.5 d	30 d	4.44	4.07 × 10^−1^	30 d	FBTR (2.45 × 10^15^)
		200 d	1.08 × 10^1^	7.03 × 10^−1^	180 d	CEFR (2.5 × 10^15^)
^32^P	14.3 d	90 d	7.55 × 10^2^	4.85 × 10^2^	80 d	CEFR (2.5 × 10^15^)
		28 d	1.00 × 10^1^	2.92 × 10^1^	28 d	HFETR (1.1 × 10^14^)
^64^Cu	12.7 h	3 d	8.95 × 10^1^(^nat^ZnO) 2.14 × 10^2^ (Enriched- ^64^Zn)	5.90 × 10^2^ (Enriched- ^64^Ni)	8 h	Low-energy proton cyclotron (11.4 MeV, 30 μA)
^67^Cu	2.58 d	10 d	1.92 (^nat^ZnO) 4.05 × 10^1^ (Enriched- ^67^Zn)	3.80 (Enriched-^67^Zn)	24 h	Medium-energy proton cyclotron (70.0 MeV, 30 μA)
				1.13 (Enriched- ^68^Zn)	53.5 h	40 MeV e-LINAC
^99^Mo	66 h	8 d	2.34 × 10^2^ (^nat^Mo)	5.44 × 10^1^ (^nat^Mo)	8 d	HFETR (1.1 × 10^14^)
			6.19 × 10^2^ (Enriched-^98^Mo)	2.04 × 10^2^ (Enriched-^98^Mo)		
			8.36 × 10^2^ (Enriched-^100^Mo)	1.48 × 10^2^ (Enriched-^98^Mo)	24 h	Electron accelerator (60 MeV, 2 mA)

## Data Availability

The data presented in this study are available on request from the corresponding author.
